# Atlanta Medical College Clinic

**Published:** 1878-11

**Authors:** 


### ATLANTA MEDICAL COLLEGE CLINIC.

---

**Service of Prof. J. G. WESTMORELAND.**

---

November 4, 1878.

Case 1.—Coran R., white male, aet. 25 years, a farmer by occupation.

You perceive, gentlemen, that this young man's general appearance does not denote very serious illness at present; nor do we find the usual evidences of acute disease, such as fever, debility, etc. Some general symptoms, however, are very prominent. The cough, which we find pretty constant since his appearance before us, denotes diseased condition of some portion of the respiratory apparatus, and his report of its continuance for some time past shows a permanence of the lesion requiring decided measures for its relief. The general symptom of pain he describes, seems to be that kind we would expect from irritation or inflammation of the bronchial mucous membrane, differing from pleural pain very materially. While the suffering of bronchitis is described as a burning sensation as of rawness, during the rapid passage of air along the tubes in the act of coughing, pleuritic pain is sharp, lancinating as though a knife was piercing the chest, and is aggravated by movement in the act of breathing, coughing, etc., and by pressure on the intercostal spaces over the inflamed pleura.

Besides these general evidences of disease, the progress of science furnishes the student at this day, means of diagnosis unknown to the profession half a century ago. Physical signs of pectoral disease, practiced by Laenec, and improved upon by Flint and others more recently, make diagnosis comparatively easy and certain. The scalpel of the ancients did not more certainly reveal the true pathological state than do now the physical means of diagnosis. The stethescope and pleximiter, in hands experienced with them, make known the nature and location of lesions connected with the thoracic viscera, which cannot otherwise be revealed during life.

In this case, for the purpose of ascertaining more certainly the cause of cough and other general symptoms of disease, I proceeded to auscultate the voice and respiration, and to percuss both sides of the chest. No dullness, indicating decided obstruction to the ingress of air is discovered in any portion of the lungs. The sounds of the voice and respiration are natural, or nearly so, except at the base of the right lung a little dullness and slight crepitation or fine bronchial rale are perceptible.

Taking these physical signs with the general symptoms of cough and sense of rawness in the chest, we locate the trouble in the base of the right lung, principally in the broncnial tubes of that part. The history given by the patient, to the effect that the frequent tickling cough, with the occasional expectoration of mucous, has troubled him for two or three weeks, and that the previous tendency has been to persistent cough after taking cold, proves the existence of chronic irritation of the bronchia.

In order to arrest this tendency to permanent chronic inflammation, counter irritation and the action of local alteratives or catheretics are advised.

To accomplish the first, croton oil is directed to be rubbed upon the front of the chest twice a day until pustulation is had, and re-applied when the pustules disappear. In order to affect directly the diseased mucous membrane of the bronchia, various local alteratives would answer the purpose if they could be made to reach the part affected. Nitrate of silver, chloride of zinc, or sulphate of copper can be used only in the forms of liquid and solid, and in these forms cannot be applied to the minute bronchia. It is true a solution of nitrate of silver may be injected into the air passages, but the result would be anything but desirable. Twenty years ago a thorongh test was made, but the sad experience of such bold experimenters is left as a warning to enterprising practitioners in future. Powdered substances cannot, of course, be made to pass over moist surfaces and reach points so remote; nor can the vapor of water or other liquids be made to carry substances not volatile in themselves. Remedies only, therefore, which may readily assume the form of fumes, can be successfully applied to the bronchial mucous membrane. Unfortunately, there are but few of these which have local alterative or catheretic property. Iodine is perhaps the only one that can be relied on for this action.

In addition, then, to the counter-irrition by croton oil, I direct that iodine be inhaled once a day for a week, and then once in two or three days for a few weeks, or until the evidences of bronchial irritation disappear. In order to protect the patient against the danger of return, it is advised that inhalations be renewed on the appearance of cough following the contraction of cold during the winter.

A very simple apparatus is sufficient for the purpose. A small vial with large mouth—a common morphine vial—may be heated on a stove or over a lamp. Into this three to six centigrams (one-half to a grain) of iodine should be placed, and the fumes inhaled at once from the vial through the mouth.

The inhalation may be made through the nasal passages, but unless these are also the subject of disease, unnecessary pain is produced in the sensitive membrane lining them, and therefore the mouth is preferable in this case.

Case 2—Anna A., mulatto, æt. 24 years, married, and the mother of three children, the youngest nine months of age. Has cough and yellow expectoration at night. Has not breathed easily through the nose for several years. In this case, auscultation of the respiration reveals the sound of rough breathing, as it is sometimes called, dependent on increased amount of mucus in various parts of the bronchia. Percussion gives normal sounds. The patient also states that lumps resembling scabs are discharged from the back part of the nose into the throat.

This, gentlemen, is a representative case of that class known as nasal catarrh, the study of which will be found more important to you than any disease I shall be able to present during the session. It is important, not so much from the danger of death attending it, but on account of its extensive prevalence and the difficulty of permanent cure.

Here we have nasal and bronchial catarrh. Commencing, perhaps, in the nostrils, it has extended into the fauces and thence to the larynx. Even when confined to the nares much difficulty is found in effecting a permanent cure, if of long duration before treatment is commenced.

The term catarrh is applicable to discharge from the nostrils, or other mucous membranes, of more than the usual quantity of mucus from any cause. Common bad cold or influenza is accompanied with profuse discharge from the nose on account of congestion of the pituitary mucous membranes, and is generally called acute catarrh.

The bladder, uterus, etc., are said to be affected with catarrh when so diseased as to keep up profuse mucus discharge. Chronic and acute are terms which denote the duration of such discharge. Chronic nasal catarrh is the name given to chronic inflammation of the nasal mucous surface, and, like chronic diseases of other parts, requires longer or shorter time for cure, in proportion to the duration. Hence, a case which has existed for several years must necessarily tax the patience of physician and subject for a long time before a cure can be effected permanently.

Another disease of the nares which is sometimes confounded with chronic inflammation, is known as ozæna, and depends upon ulceration or caries of the bones just beneath and on the lateral walls of the nares. This differs materially from catarrh, and seldom if ever results from simple inflammation of the mucous membrane. The ulceration of these bones of the nose may depend upon syphilitic contamination or strumous diathesis, or, as is often the case, occurs without constitutional taint of any kind or other known cause. This loathsome affection is the dread of persons affected with catarrh, but for their consolation the physician can generally deny such probable result.

The treatment of chronic nasal catarrh, or, more properly speaking, chronic inflammation of the pituitary membrane, is necessarily tedious when the disease is of long standing; and in order to succeed, must be continued at proper intervals for months and even years. The tendency to return always exists, and without vigilance on the part of patient and physician, the disease, after having apparently disappeared, will be found to assume its original severity in a few months.

The nasal douche of salt water or other medicated liquid, used daily, is the common treatment for such cases. The following will be used in this case for a few days:

℞.—Sulphate zince, . . . . . 1 gm.
Water, . . . . . . . 50 cg.

Dissolve.

To be placed in a douche bottle and elevated so as to flow through a rubber tube into one nostril and out at the other, thus coming in contact with the entire surface of each.

